# Is the ASA physical status classification system a good prognostic index for ICU admissions?

**DOI:** 10.1186/cc9882

**Published:** 2011-03-11

**Authors:** A Gregório, A Pais-de-Lacerda, C França

**Affiliations:** 1 Hospital de Santa Maria, Lisbon, Portugal

## Introduction

The physical state of the patient before surgery is defined by the American Society Anesthesiology (ASA) physical status classification system. The Simplified Acute Physiology Score (SAPS II) provides an estimate for the risk of intrahospital death for ICU patients. The Sequential Organ Failure Assessment (SOFA) score is used to monitor the patient's condition during his/her stay in the ICU, assessing the extent of organ dysfunction or failure. Is the ASA physical status classification system a good prognostic index for determining postsurgical patient's admittance to the ICU? What is the evolution of these patients? Could we predict the outcome of these patients?

## Methods

A retrospective analysis of the ASA, SAPS II and SOFA of all postsurgical patients admitted to an ICU, between 1 May and 31 October 2010.

## Results

Total ICU admissions: 323 patients, 118 being postsurgical patients (mortality: 12 patients - 10.17%). Maximum patient SOFA: between 0 and 19. Patient SAPS II: between 8 and 99. Of the 118 patients, five had ASA 5, a mortality of 100% being expected but only three died. The expected mortality rate of the three deceased (SAPS II: 58, 99, 80) was 5.2%, 92.5%, 98.4%, respectively. The two patients who got better had a SAPS II of 21 and 56 with a maximum SOFA of 4 and 16, which means that they improved significantly, against all odds. Most ICU admitted patients were ASA 3 and ASA 4. Fifty per cent of ASA 3 patients presented a maximum SOFA between 0 and 5; maximum SOFA was higher in 34% of ASA 3 patients (5 to 10) with predicted ICU mortalities of up to 7% and 46%, respectively. Four patients of the ASA 3 group died. Of the ASA 4 patients, 43% had a maximum SOFA between 5 and 10, and 34% presented a lower maximum SOFA (0 to 5). In 10 (26%) ASA 4 patients, maximum SOFA exceeded 11 with a mortality ICU predicted rate of 56%. In fact, five died. The reason for admission to the ICU of the 20 patients with lower ASA (17 ASA 2 patients, three ASA 1 patients) was a need for tighter monitoring or stabilization of postsurgical complications. Indeed, all deaths in the ASA 2 (1/17) and ASA 3 (4/38) groups were related to complications from co-morbidities.

## Conclusions

ASA 3 and ASA 4 patients are those who benefit the most from a stay in an ICU, enabling one to reduce mortality predicted by SAPS II and SOFA scores. The ASA physical status classification system is not a good indicator of mortality, but its association with SAPS II and maximum SOFA scores define more effectively the severity and prognosis of the postsurgical patient.
